# Silica/quercetin sol–gel hybrids as antioxidant dental implant materials

**DOI:** 10.1088/1468-6996/16/3/035001

**Published:** 2015-05-05

**Authors:** Michelina Catauro, Ferdinando Papale, Flavia Bollino, Simona Piccolella, Sabina Marciano, Paola Nocera, Severina Pacifico

**Affiliations:** 1 Department of Industrial and Information Engineering, Second University of Naples, Via Roma 29, 81031 Aversa, Italy; 2 Department of Environmental, Biological and Pharmaceutical Sciences and Technologies, Second University of Naples, Via Vivaldi 43, 81100 Caserta, Italy

**Keywords:** sol–gel technique, quercetin, antioxidant activity, bioactivity, biocompatibility

## Abstract

The development of biomaterials with intrinsic antioxidant properties could represent a valuable strategy for preventing the onset of peri-implant diseases. In this context, quercetin, a naturally occurring flavonoid, has been entrapped at different weight percentages in a silica-based inorganic material by a sol–gel route. The establishment of hydrogen bond interactions between the flavonol and the solid matrix was ascertained by Fourier transform infrared spectroscopy. This technique also evidenced changes in the stretching frequencies of the quercetin dienonic moiety, suggesting that the formation of a secondary product occurs. Scanning electron microscopy was applied to detect the morphology of the synthesized materials. Their bioactivity was shown by the formation of a hydroxyapatite layer on sample surface soaked in a fluid that simulates the composition of human blood plasma. When the potential release of flavonol was determined by liquid chromatography coupled with ultraviolet and electrospray ionization tandem mass spectrometry techniques, the eluates displayed a retention time that was 0.5 min less than quercetin. Collision-activated dissociation mass spectrometry and untraviolet-visible spectroscopy were in accordance with the release of a quercetin derivative. The antiradical properties of the investigated systems were evaluated by DPPH and ABTS methods, whereas the 2,7-dichlorofluorescein diacetate assay highlighted their ability to inhibit the H_2_O_2_-induced intracellular production of reactive oxygen species in NIH-3T3 mouse fibroblast cells. Data obtained, along with data gathered from the MTT cytotoxicity test, revealed that the materials that entrapped the highest amount of quercetin showed notable antioxidant effectiveness.

## 1. Introduction

In recent years, research in the field of biomaterials endowed with high bioactivity and biocompatibility has given increased attention to the preparation of dental and/or orthopedic devices and implants [1–3]. New synthetic strategies have been developed to define a broad and durable application of these devices, which respond well to a more demanding request by the end user to combine the concepts of health and aesthetics, especially in the field of dentistry [4–7].

The promising chemical and physical properties of different biomaterials have been widely investigated [8, 9], and detailed knowledge about their potential toxicities has also been provided [10–14]. Nevertheless, diseases resulting from failures of an implant system are still continuously reported. An example is peri-implantitis, which takes hold in the oral cavity as a result of a multifactorial process where the dominant role seems to be the overproduction of reactive oxygen species (ROS) [15]. At the cellular level, this imbalance between the production of reactive chemical species with radical and nonradical natures causes an oxidative stress condition. Oxidative stress is defined as an uncontrolled process [16] by which the massive production of reactive species is conjugated to the damage they can cause to biological molecules (e.g., DNA, proteins, carbohydrates, and lipids). The sustained damage by ROS results in harmful consequences at the cellular, tissue, and systemic levels. It has been broadly demonstrated that this condition is involved in the establishment of some chronic and degenerative diseases (e.g., cancer and neurodegenerative diseases), cardiovascular diseases, and in oral cavity pathologies such as periodontitis. As for periodontal disease [17], peri-implantitis is triggered by gram-negative, anaerobic, or microaerophilic bacteria and by the establishment of an inflammation outbreak capable of inducing the formation of ROS. Once formed, the ROS stimulate the production of proinflammatory cytokines, thus triggering a vicious circle [18]. Some evidence suggests that the total antioxidant capacity of saliva undergoes a significant decrease in the peri-implantitis condition; the concentration of uric and ascorbic acids—the main metabolites with antioxidant activity—appears to be strongly reduced [19]. In this context, biomaterials research that can counter the damaging effects of oxidative stress could represent a valuable and useful tool for therapies that target these medical problems. Indeed, the hypothesis of counteracting the onset of peri-implantitis and other dental diseases with suitable antioxidant supplementation is already one of the objectives of modern toxicological research in the biomaterials field [20, 21]. The addition of N-acetyl cysteine in resin-based materials has favored the formulation of a new material in which the intrinsic cytotoxicity of the resin was potentially detoxified [22]. Similarly, the use of Trolox^®^ seems to reduce the cytotoxicity induced by biomaterials [23]. Implants were developed using antioxidant stabilizing compounds such as vitamin E, which was typically added in low concentrations during ethylene polymer consolidation. Clinical data suggest that blended formulations with concentrations of less than 0.1% vitamin E maintain the same physical and mechanical properties and can prevent oxidation for up to 24 months post-implantation [24]. Recently, antioxidant polymers, which are molecules that have natural antioxidants (quercetin and curcumin) incorporated into a polymer backbone, were synthesized to attenuate material-induced oxidative stress [25]. The preparation of silica microspheres through a novel method that uses a polyol-in-oil-in-water (P/O/W) emulsion and sol–gel methods as techniques for stabilizing quercetin has been also reported [26].

In order for new biomaterials with native intrinsic antioxidant properties to be suitable and widely used in the clinical setting, they should provide the benefit of relatively high antioxidant content and continuous local antioxidant potential while the implant is present. In the search for new biocompatible biomaterials that provide antioxidant functionality and do not exacerbate the body’s normal oxidant and inflammatory response, our research group has optimized the synthesis of novel, intrinsically antioxidant, quercetin-based biomaterials, which could be used in dentistry as components in glass ionomer cement and in medicine as replacements for bone implants. Quercetin is a naturally occurring flavonol broadly recognized for its antioxidant and anti-inflammatory properties [27]. We applied the sol–gel technique for this purpose. The sol–gel process is currently one of the most studied and used techniques for the production of high-quality glassy and ceramic materials [28]. Interest in this technique is stimulated by the extreme versatility of the method, which, being highly controllable, has many advantages compared to traditional methods. In recent years, we have extensively adopted sol–gel routes for the formulation of inorganic or hybrid inorganic–organic materials that are potentially useful in the dental and orthopedic fields [29–33].

The addition of quercetin (in weight percentages of 5, 10, or 15%) to an inorganic silica matrix in the synthetic process was aimed at formulating an antioxidant, bioactive, and biocompatible glass material in which the natural molecule firmly integrates into the inorganic network. Liquid chromatography coupled with electrospray ionization tandem mass spectrometry (LC-ESI/MS/MS) techniques were applied to study the antioxidant drug release. Ultraviolet–visible (UV–vis) and collision-activated dissociation (CAD) mass spectrometry (MS) techniques were applied to define the integrity of the quercetin skeleton. *In vitro* bioactivity of synthesized materials, which is indicative of their osseointegration ability, was investigated by soaking the samples in a simulated body fluid (SBF) and using scanning electron microscopy (SEM) to observe the hydroxyapatite formation on the surface. Since one of the recommended and appropriate steps for the biological assessment of medical devices is the *in vitro* assessment of cytotoxicity of new biomaterials, an MTT assay was carried on the NIH-3T3 murine fibroblast cell line. The ability of the new system to exhibit antioxidant properties was evaluated first by applying DPPH and ABTS methods, and then by a 2,7-dichlorofluorescein diacetate assay on NIH-3T3 mouse fibroblast cells.

## 2. Materials and methods

### 2.1. Sol–gel synthesis

Inorganic SiO_2_ and SiO_2_/quercetin hybrids (Si/Que) that differed in their drug content (5, 10, and 15%wt) were prepared by means of a sol–gel process (table 1).

**Table 1.** Composition and label of the synthesized systems.

Label	Quercetin amount in the SiO_2_ matrix (wt%)
SiO_2_	0%
Si/Que5	5%
Si/Que10	10%
Si/Que15	15%

The inorganic silica gel was synthesized by adding tetraethyl orthosilicate (TEOS; Si(OC_2_H_5_)_4_, Sigma-Aldrich) to a solution containing HNO_3_ (≥65%, Sigma-Aldrich) and distilled water in ethanol 99.8% (Sigma-Aldrich). The acidic environment favors the kinetics of hydrolysis and condensation reactions, and thus affects the microstructural properties of the inorganic matrix [34]. The structural characteristics were also influenced by the H_2_O/alkoxide molar ratio. When the value was less than four, compact and mesoporous materials were obtained [35]. The molar ratio used for the reagents was TEOS:HNO_3_:EtOH:H_2_O=1:1.7:6:2.

To prepare the hybrid materials, quercetin (Sigma-Aldrich) was dissolved in ethanol and added to the synthesized silica sol under stirring. After gelation, the products were air-dried at 50 °C for 24 h to remove the residual solvent.

### 2.2. Characterization studies

The characterization of the developed materials was implemented using different techniques.

The microstructure of the synthesized gels was studied by SEM (Quanta 200, FEI, Netherlands).

Fourier transform infrared (FTIR) transmittance spectra were recorded in the 400–4000 cm^−1^ region using a Prestige 21 (Shimadzu, Japan) system, equipped with a deuterated tryglycine sulphate detector with potassium bromide windows, with a resolution of 2 cm^−1^ (45 scans). KBr pelletized disks containing 2 mg of each sample and 198 mg of KBr were made. The FTIR spectra were processed by Prestige software (IR solution).

The UV–vis spectra of extracts from discs of each different synthesized material, which had previously been soaked in Dulbecco’s Phosphate Buffer Saline (DPBS) SBF, were acquired in the range 200–600 nm using a Shimadzu 1700 spectrophotometer.

Mass spectra were recorded using a Quattro Micro™ triple quadrupole mass spectrometer (Waters/Micromass, Manchester, UK) equipped with an electrospray ionization (ESI) source operating in the positive ion mode. Nitrogen was used as the nebulizer and solvent gas at flow rates of 50 and 500 l/h, respectively. Source and dissolution temperatures were set at 120 °C and 350 °C, respectively. Applied potentials of the electrospray capillary and of the cone were set at 2.50 kV and 10 V, respectively. CAD mass spectra were recorded by introducing argon as a collision gas into the RF-only quadrupole at a pressure of ∼3.0 × 10^−3^ mbar to minimize multiple collisions. The mass range was recorded from 20 to 800 m z^−1^, with interchannel and interscan delays of 0.02 s and 0.1 s, respectively. The collision energy used for CAD analysis was 10 eV (E_lab_). Data acquisition and processing were carried out using the software MassLynx™ version 4.0 supplied with the instrument.

### 2.3. *In vitro* bioactivity

The *in vivo* bone-bonding ability of the synthesized materials was evaluated by an *in vitro* apatite forming-ability test. Pelletized disks of SiO_2_ and Si/Que hybrids were soaked for 7, 14, and 21 days in an SBF with an ion concentration nearly equal to that of human blood plasma [36]: Na^+^ 142.0, K^+^ 5.0, Ca^2+^ 2.5, Mg^2+^ 1.5, Cl^−^ 147.8, HCO_3_^−^ 4.2, 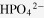 1.0, SO_4_^2−^ 0.5 mM. Polystyrene bottles containing the samples were placed in a water bath, and during soaking the temperature was kept fixed at 37 °C. Taking into account that the ratio between the total surface of the material exposed to the SBF and its volume influences the reaction of the hydroxyapatite layer formation, a constant ratio of 10 mm^2^ ml^−1^ of solution was respected [37]. Moreover, the SBF solution was changed every 2 days to avoid depletion of the ionic species in the SBF due to the formation of biominerals by the samples.

After each soaking period in the SBF, the samples were dried in a desiccator and then subjected to SEM combined with energy-dispersive x-ray spectroscopy (EDS) to evaluate their ability to form an apatite layer on their surfaces.

### 2.4. *In vitro* release test

Three discs of each quercetin silica-based material (Si/Que5, Si/Que10, and Si/Que15; 200.0 mg each) were soaked in 7.5 ml DPBS SBF at 37 °C under continuous stirring. LC/UV-ESI/MS analyses were carried out for up to 7 days.

The chromatographic apparatus consisted of an Alliance 2695 separations module equipped with a column heater and a sample chiller and a Waters 2487 dual-wavelength UV detector. Separations were achieved using a Synergy Hydro^®^ C8 reversed-phase column (4.0 *μ*m particle size, 250 × 4.6 mm i.d., Phenomenex) at a flow rate of 0.40 ml min^−1^ with isocratic elution of 0.1% (v/v) formic acid aqueous solution and acetonitrile (1:3, v/v). The UV detection of quercetin was performed at 380 nm. To generate the calibration curve, several known concentrations of the standard (0–2.0 mM) were injected; the response factors based on the linear regression of a plot of peak area versus concentration were computed. The injection volume was 10 *μ*l. The liquid chromatography (LC) system was coupled to the previously described Quattro Micro™ triple quadrupole mass spectrometer (Waters/Micromass, Manchester, UK).

### 2.5. Determination of DPPH^•^ scavenging capacity

To estimate the 2,2-diphenyl-1-picrylhydrazyl (DPPH^•^) scavenging capability, extracts from the investigated matrices (50.0 *μ*l) were added to a DPPH^•^ methanol solution (9.4 × 10^−5^ M; 1.0 ml final volume) at room temperature. After 30 min, the absorption at 515 nm was measured by a Shimadzu UV-1700 spectrophotometer in reference to a blank. The results were expressed in terms of the percentage decrease of the initial DPPH^•^ radical absorption by the test samples [38].

### 2.6. Determination of ABTS^•+^ scavenging capacity

The determination of ABTS^•+^ solution scavenging capacity was estimated as previously reported [38]. 2,2′-azinobis-(3-ethylbenzothiazolin-6-sulfonic acid (ABTS) radical cation was generated by reacting ABTS (7.0 mM) and potassium persulfate (2.45 mM). The mixture was allowed to stand in the dark at room temperature for 12–16 h. Thus, the ABTS^•+^ solution was diluted with DPBS (pH 7.4) in order to reach an absorbance of 0.70 at 734 nm. Extracts from investigated matrices (50.0 *μ*L) were added in diluted ABTS^•+^ solution (1.0 ml final volume). After 6 min of incubation, the absorption at 734 nm was measured by a Shimadzu UV-1700 spectrophotometer in reference to a blank. The results were expressed in terms of the percentage decrease of the initial ABTS^•+^ absorption by the test samples.

### 2.7. Cell culture and cytotoxicity assessment

Cytotoxicity was measured through an MTT [3-(4,5-dimethyl-2-thiazolyl)-2,5-diphenyl-2 H-tetrazolium bromide] cell-growth inhibition assay (indirect test) using the NIH-3T3 murine fibroblast cell line. Investigated extracts were obtained from discs of the studied materials, which were previously immersed for 24 h in 3.5 ml of a complete culture medium at 37 °C under continuous stirring.

The cell line, purchased from American Type Culture Collection, was grown in Roswell Park Memorial Institute (RPMI) medium 1640 high-glucose medium supplemented with 10% fetal bovine serum, 50.0 U ml^−1^ penicillin, and 100.0 *μ*g ml^−1^ streptomycin at 37 °C in a humidified atmosphere containing 5% CO_2_.

The NIH-3T3 cell line was seeded in 96-multiwell plates at a density of 1.0 × 10^4^ cells/well. After 24 h of incubation, cells were treated with 200 *μ*l culture medium solution containing 50 *μ*l of extracts from silica and silica-quercetin materials (with entrapped quercetin at 5, 10, and 15%wt). At 24, 48, and 72 h of incubation, cells were treated with 150 *μ*l of MTT (0.50 mg ml^−1^) dissolved in the culture medium for 1 h at 37 °C in a 5% CO_2_ humidified atmosphere. The MTT solution was then removed, and 100 *μ*l of dimethyl sulfoxide was added to dissolve the originated formazan. Finally, the absorbance of each well at 570 nm was determined using a Tecan SpectraFluor fluorescence and absorbance reader. Cell viability was expressed as percentage of mitochondrial redox activity of the cells treated with the extracts compared to an untreated control [39]. Tests were carried out performing twelve replicate (*n* = 12) measurements for three samples of each extract (in total: 12 × 3 measurements).

### 2.8. Measurement of intracellular ROS formation

The levels of intracellular ROS were determined by the change in fluorescence resulting from the oxidation of the fluorescent probe, 2′,7′-dichlorofluorescein diacetate (DCFH-DA). When applied to intact cells, DCFH-DA readily diffuses through the cell membrane and is hydrolyzed enzymatically by intracellular esterases to nonfluorescent DCFH. In the presence of ROS, DCFH is oxidized to highly fluorescent DCF, whose fluorescent intensity is proportional to the amount of ROS formed intracellularly [40].

The NIH-3T3 cell line, seeded in 96-multiwell plates at a density of 1.0 × 10^4^ cells/well, was incubated with DCFH-DA (10 *μ*M) in DPBS for 30 min. At the end of incubation, the DCFH-DA solution was removed and cells were cotreated with Que15 or Si/Que15 materials (50 *μ*l) and H_2_O_2_ (400.0 *μ*M). The fluorescence intensity was measured at 485 nm excitation and 535 nm wavelength in a Tecan SpectraFluor fluorescence and absorbance reader. Results were expressed as percentages relative to oxidized cell lines arbitrarily set at 100%.

## 3. Results and discussion

### 3.1. Characterization of synthesized materials

All the synthesized materials appeared transparent, glassy, and reddish in color. They were characterized by the same morphology. Their compact structure could be due to the H_2_O/alkoxide molar ratio used. In fact, it is known that when the H_2_O/alkoxide molar ratio has a value less than 4, compact and mesoporous materials are obtained [35].

**Figure 1.**
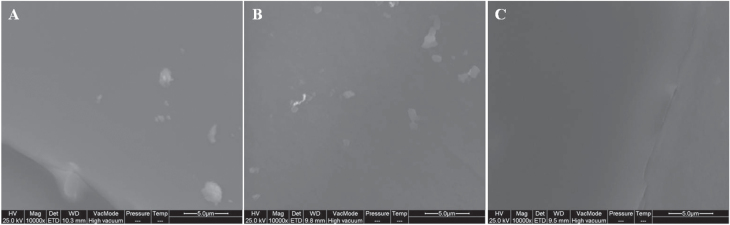
SEM micrographs of: (a) Si/Que5, (b) Si/Que10, and (c) Si/Que15.

Figure 1 shows SEM micrographs of the Si/Que systems. No appreciable difference in surface morphology could be observed. The organic and inorganic phases were indistinguishable, thus confirming the hybrid feature of the synthesized sol–gel materials.

Information on the structural organization of the bioactive molecule and silica matrix within the synthesized materials was determined by FTIR spectroscopy.

**Figure 2.**
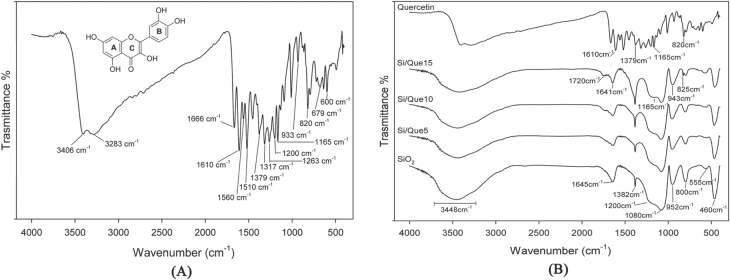
FTIR spectra of (a) pure quercetin, (b) Si/Que hybrid systems. Spectra are vertically shifted for clarity.

The FTIR spectrum for pure quercetin is shown in figure 2(a), where its characteristic bands [41] were detected. OH groups stretching were detectable at 3406 and 3283 cm^−1^, whereas OH bending of the phenol function was detectable at 1379 cm^−1^. The C=O aryl ketonic stretch absorption was evident at 1666 cm^−1^. C=C aromatic ring stretch bands were detectable at 1610, 1560, and 1510 cm^−1^. The in-plane bending band of C–H in aromatic hydrocarbon was detectable at 1317 cm^−1^, and out-of-plane bending bands were evident at 933, 820, 679, and 600 cm^−1^. Bands at 1263, 1200, and 1165 cm^−1^ were attributable to the C–O stretching in the aryl ether ring, the C–O stretching in phenol, and the C–CO–C stretch and bending in ketone, respectively.

To identify the interaction between the quercetin and the SiO_2_ matrix, the FTIR spectra of pure quercetin (Que) and quercetin-free silica (SiO_2_) were compared to those of the quercetin-entrapped silica based-materials (Si/Que), as shown in figure 2(b).

In the SiO_2_ spectrum (figure 2(b)), all typical peaks of the silica sol–gel materials [42–44] were visible. The strong band at 1080 cm^−1^ with a shoulder at 1200 cm^−1^, and the peaks at 800 cm^−1^ and 460 cm^−1^, were ascribed to asymmetric and symmetric Si-O stretching motions and the bending Si-O-Si mode, respectively. The band at 952 cm^−1^ was assigned to Si-OH bond vibrations [45] and the low-intensity band at 555 cm^−1^ was attributed to residual four-membered siloxane rings in the silica network [42, 46–49]. Moreover, the 1382 cm^−1^ sharp peak was due to N–O stretching of residual nitrate anions resulting from HNO_3_ used as catalyst in the synthesis procedure; the broad, intense band at 3448 cm^−1^ and the peak at 1645 cm^−1^ were due to OH stretching and bending vibrations in the hydration water. The position and the shape of the latter bands suggest the presence of hydrogen-bonded solvent molecules (H_2_O) and hydrogen-bonded OH groups attached to the Si atoms [50].

All discussed peaks were also visible in the Si/Que materials spectra (figure 2(b)). However, the drug addition caused a slight decrease in their intensity and the down-shift of the bands of Si-OH, which appeared at 943 cm^−1^. These changes could be due to interactions between the inorganic matrix and the quercetin, which caused variations in bond length, and thus of bond strength, in the SiO_2_ network. In particular, the down-shift of the Si–OH signal, together with the broadening of the OH band at 3448 cm^−1^, suggested the formation of H-bonds, which involved the hydroxyl groups of the silica matrix. These findings were confirmed by the observation that in these spectra, some typical peaks of quercetin were also detectable in a flavonol concentration-dependent manner. In particular, OH bending of the phenols band at 1379 cm^−1^ appeared broadened and had a higher intensity in the spectra of the hybrid materials, so that it overlapped with the nitrate peak. The C–CO–C stretch and bending in the ketone band also appeared to be slightly broadened at 1165 cm^−1^. The slight shift, the broadening, and the increase in terms of energy absorption of the OH bending band and the slight broadening of the ketone band suggested that quercetin was associated with silica matrix OH groups through hydrogen bonds that involved the OH of phenols and the C=O of the C ring. Changes were detected in the region of the double bond-related absorptions, where the bands at 1720 and 1641 cm^−1^ were evident. The weak intensity of the first band (absent in both the quercetin and the SiO_2_ spectrum) allowed us to hypothesize that structural modification on the flavonol C-ring was a consequence of the oxidation of the *γ*-pyrone ring in the molecule (ring C). The conjunction loss among the aromatic B-ring and the C-ring consisted of the up-shifts of the C=C aromatic ring stretching band at 1610 cm^−1^ at a higher absorption value (1641 cm^−1^). Aromatic out-of-plane C-H bending vibrations at 820 and 793 cm^−1^ were also up-shifted at 825 and 797 cm^−1^, respectively.

**Figure 3.**
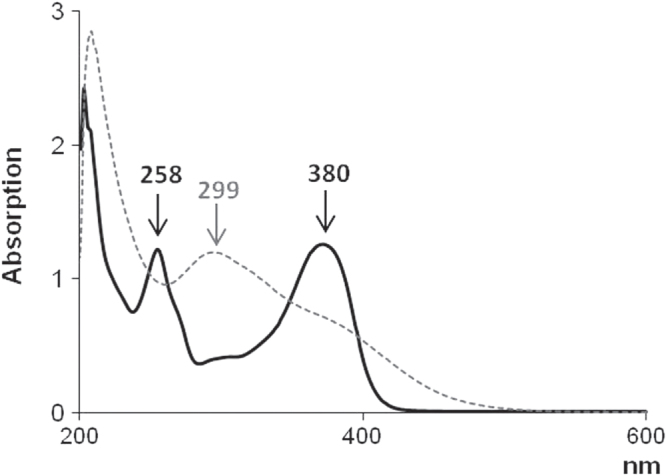
UV–vis spectra of quercetin (solid black curve) and extracts from quercetin-entrapped silica materials (dashed gray curve).

To confirm the structural modification of the quercetin skeleton, UV–vis spectroscopic analysis was equally performed on pure quercetin dissolved in DPBS and extracts from the discs of quercetin-entrapped silica materials previously soaked in DPBS for 1 h (figure 3). Quercetin exhibited two absorption bands at 258 and 380 nm. The first one was considered to be associated with absorption due to the B-ring cinnamoyl system, and the second with absorption involving the A-ring benzoyl system [51]. Upon sol–gel synthesis, a new absorption band appeared around 299 nm; the absorption band at 380 nm became less pronounced, and at 258 nm it disappeared. This absorption spectrum was in agreement with the reported absorption spectrum of 2-(3′,4′-dihydroxybenzoyl)-2,4,6-trihydroxybenzofuran-3(2H)-one [51], a quercetin-oxidized derivative commonly produced in aqueous solution and oxygen-free conditions. This finding allowed us to hypothesize that the oxidation process was achieved when sol–gel synthesis was performed and promoted by acidic condition and ethanol presence.

**Figure 4.**
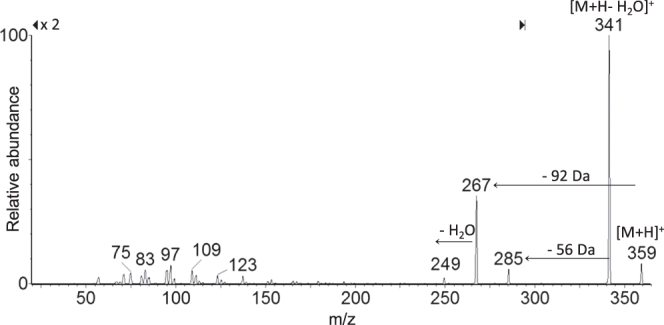
MS/MS spectrum of the protonated quercetin derivative at m/z 359.

**Figure 5.**
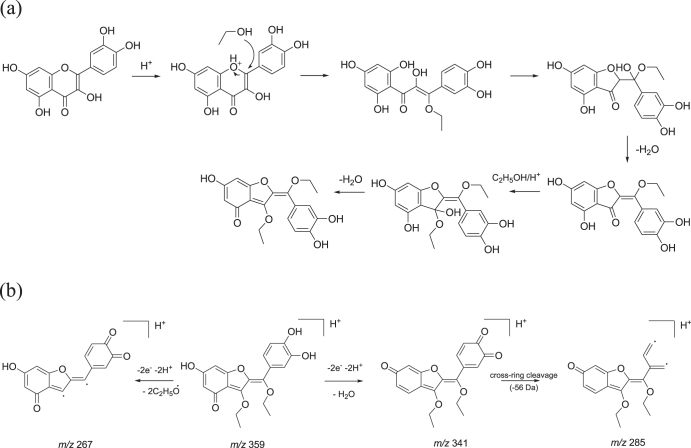
(a) Proposed synthesis pathway of the tentatively identified quercetin derivative. (b) Proposed fragmentation pattern of the tentatively identified quercetin derivative ([M + H]^+^ at m/z 359).

**Figure 6.**
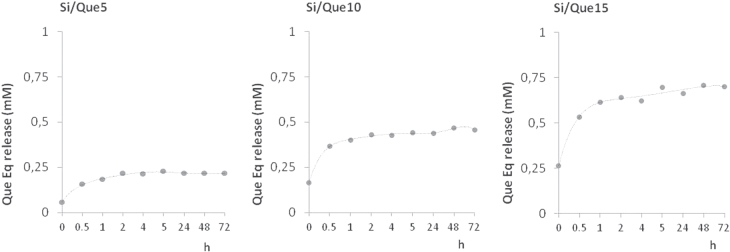
Quercetin derivative release from Si/Que5, Si/Que10, and Si/Que15. Values, expressed as quercetin equivalents (Que Eq, mM), are the mean ±standard deviation (SD) of measurements carried out on three samples of each synthesized material analyzed three times.

When the potential release of the flavonol was addressed applying LC-ESI/MS/MS analyses on extracts obtained from discs of each investigated material, a slow and steady release of one single product with a retention time 0.5 min less than pure quercetin was recorded. The structural identity of the quercetin derivative was elucidated through the MS technique. The MS/MS spectrum of the [M + H]^+^ ion at m/z 359 provided fragment ions at m/z 341 [M + H-H_2_O]^+^, 285, 267, and 249 (figure 4). The proposed synthesis pathway and mass fragmentation pattern of the tentatively identified molecule are shown in figures 5(a) and (b), respectively. The slow and steady release observed could be due to the high silanol content and the hydrophilic surface of the silica inorganic network, which on one side was able to increase drug loading through the hydrogen bonds’ onset, and on the other allowed drug release to slow down. The amount of drug released increased proportionally to the initial quercetin loaded. Matrix dissolution in DPBS and diffusion could play a key role in the observed release rate (figure 6).

### 3.2. Bioactivity test

**Figure 7.**
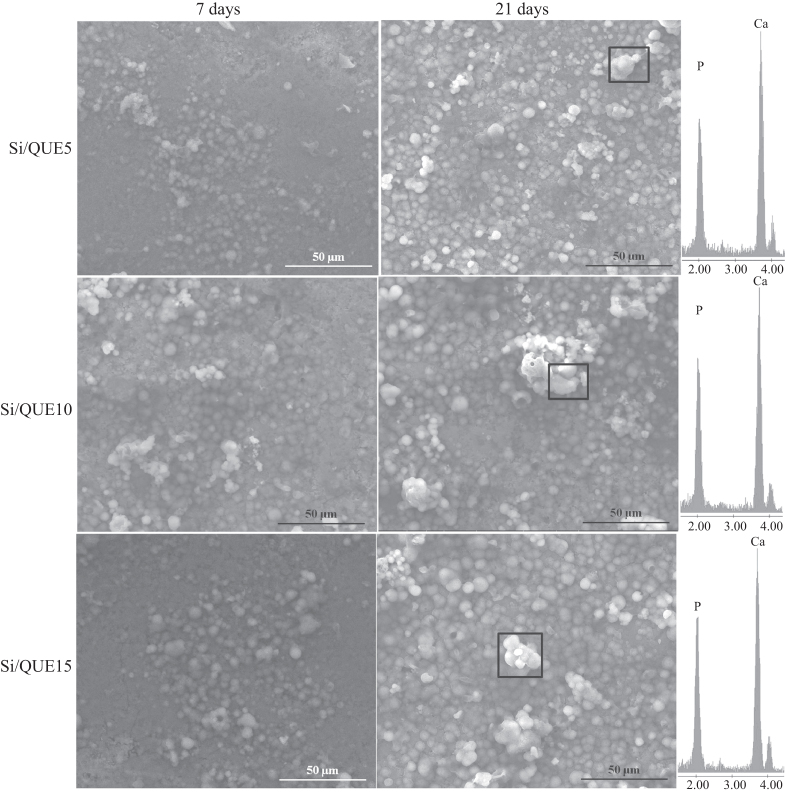
SEM micrographs of Si/Que5, Si/Que10, and Si/Que15 systems after 7 days and 21 days of soaking in SBF. EDS analysis of the globule in the box.

All samples were observed by SEM after 7, 14, and 21 days of soaking in SBF (figure 7). The formation of crystals with the typical globular shape of the hydroxyl-apatite was detected on the surface of all samples after 7 days of immersion in SBF. EDS analysis showed that the ratio between the atomic content of Ca and P of the observed globules was also 1.6 in hydroxyl-apatite [37]. The amount of those crystals increases with the time of exposure to the SBF, and they entirely cover the surface of all disks after 21 days of incubation in the solution. Any significant difference in terms of precipitate apatite amount is recorded as a function of quercetin content. Therefore, the quercetin content does not affect the bioactivity of the samples.

The formation of apatite after soaking in SBF can be explained by the presence of Si-OH groups on their surfaces [29]. These groups, combined with the Ca^2+^ ions present in the fluid, originate the increase of positive charges on their surfaces. The Ca^2+^ ions combine with the negative charge of the phosphate ions to form amorphous phosphate, which spontaneously transforms into hydroxyl-apatite [Ca_10_(PO_4_)_6_(OH)_2_] [52]. The obtained results suggest that quercetin is released in SBF, leaving the Si-OH free to interact, and it does not influence the electrostatic interactions involved in the nucleation process or the ion exchanges.

### 3.3. Antiradical properties of synthesized materials

**Figure 8.**
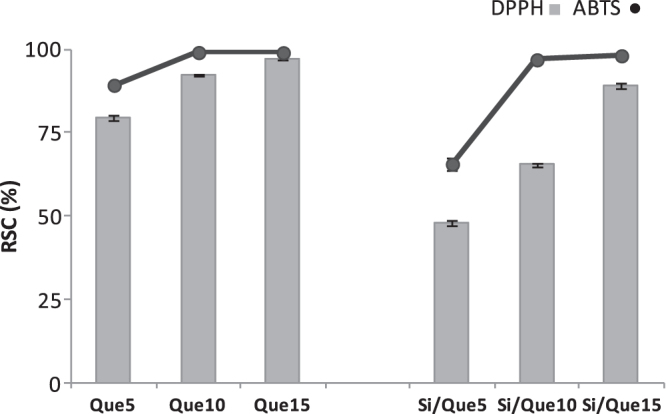
Radical scavenging capacity (RSC, %) of Si/Que5, Si/Que10, and Si/Que15, and silica-free quercetin samples (Que5, Que10, and Que15) towards DPPH^•^ radical, ABTS^•+^. Values, reported as percentage versus a blank, are the mean ±SD of measurements carried out on three samples (*n* = 3) analyzed three times.

To investigate the radical scavenging capacity of the synthesized materials, DPPH and ABTS tests were performed. Both methods are based on the use of a radical species as probe. The activity was compared to that of pure quercetin (figure 8). Although all the investigated samples massively reduced the radical species target, it was noted that for the pure molecules, the scavenging efficiency of the new materials was strongly dependent on the quercetin concentration therein.

Indeed, initial quercetin concentration seemed to play a predominant role in the antiradical capability performance. When Si/Que10 and Si/Que15 samples were tested, they were able to reduce the radical species, just as pure quercetin did. The presence of a lower dose of quercetin defined a decrease in the antioxidant power. This finding suggested that the preservation of the antioxidant properties of quercetin could be realized by adopting appropriate ratios between the concentration levels of the inorganic and organic materials. The weak antiradical capability of Si/Que5, as opposed to the Que5 scavenging efficacy, highlighted the synthesis of materials in which the flavonol underwent structural modifications. It is well known that the presence of the 2,3-double bond, the 3-hydroxyl group, and the *o*-catechol group (3′,4′-OH) are determining structural features for the high antioxidant capacity of quercetin [53]. The *o*-catechol function retention in the B-ring could explain the antioxidant data obtained.

### 3.4. Influence of SiO_2_/quercetin-synthesized materials on mitochondria and cell proliferation

To assess the influence of the extracts from the synthesized sol–gel materials on mitochondria and cell proliferation, an MTT cytotoxicity assay was performed on the NIH-3T3 murine fibroblast cell line. The cell line was treated for exposure times of 24 and 48 h with extracts obtained by placing discs of the investigated materials in a complete culture medium for 24 h.

**Figure 9.**
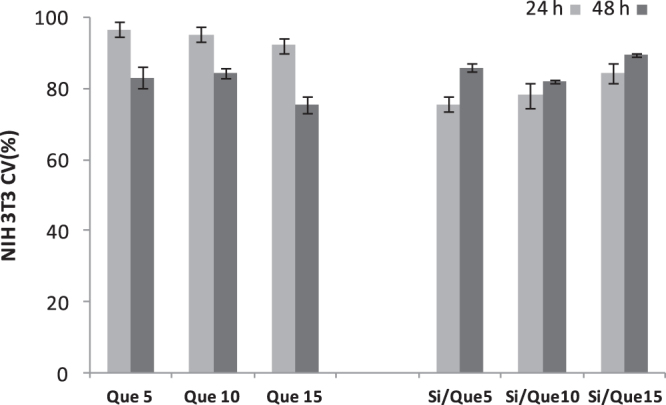
Cell viability (CV, %) toward NIH-3T3 cells of Si/Que5, Si/Que10, and Si/Que15, and silica-free quercetin samples (Que5, Que10, and Que15) at 24 h and 48 h exposure times by means of MTT test results. Values, reported as percentage versus an untreated control, are the mean ±SD of measurements carried out on three samples (*n* = 3) analyzed 12 times.

The quantitative measurement of the extracellular reduction of the yellow-colored water-soluble tetrazolium dye to insoluble formazan crystals allowed us to state that extracts obtained by the different samples affect cell viability and the proliferation of tested cells (figure 9). It was observed that all the tested samples were able to cause only mild *in vitro* suppression of cell functions to levels that would be acceptable on the basis of standards used to evaluate alloys and composites (<25% suppression of deydrogenases activity). In particular, synthesized materials showed an improved time-dependent biocompatibility. This effect was not recorded for pure quercetin, which was able to exert an antiproliferative effect at 48 h that was stronger than at 24 h.

### 3.5. Effect of Si/Que15 system on intracellular ROS generation

**Figure 10.**
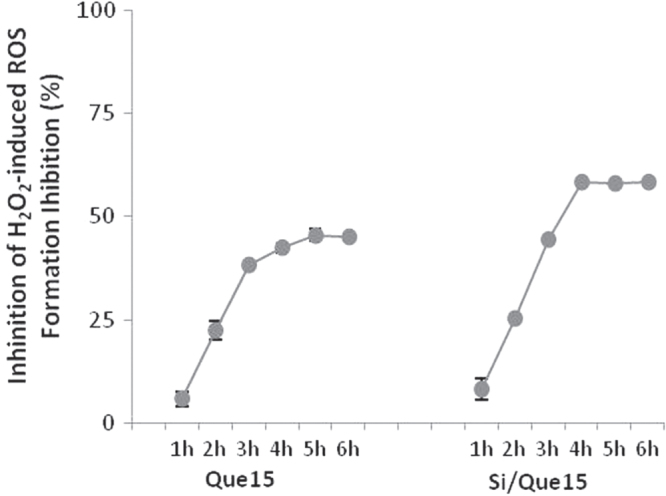
Effect of Que15 and Si/Que15 on H_2_O_2_-induced ROS generation in NIH-3T3 cells. Values, reported as percentage versus the oxidized control, are the mean ±SD of measurements carried out on three samples (*n* = 3) analyzed 12 times.

To examine whether the extracts from Si/Que15 synthesized materials could inhibit and/or prevent ROS generation induced by the known free radical generator hydrogen peroxide, the NIH-3T3 cell line was incubated with its extract and H_2_O_2_ (400 *μ*M). The assay was performed for 6 h and the DCF fluorescence formation was determined by taking data every hour. The addition of the Si/Que15 extract sample massively reduced the percentage of ROS formation in a time exposure-dependent manner. After 3 h, an inhibition of ROS formation equal to 64.0% was recorded. The antioxidant potential of the Si/Que15 extract appeared to be greater than that exerted by Que15 (figure 10), probably because of its higher bioavailability and permeability. The accessibility to lipophilic cellular membranes could explain the decrease of H_2_O_2_-induced intracellular ROS production. H_2_O_2_ is likely to amplify intracellular ROS production by generating ROS in the medium and/or cellular membranes, as well as in the intracellular matrix. Therefore, differences in the distribution in the medium/cellular membranes between quercetin and its derivative may greatly affect their effectiveness for H_2_O_2_-induced intracellular ROS production when added concurrently with H_2_O_2_ to cultured cells. Furthermore, quercetin instability and its metabolic conversion explain the reason for the lack of a significant effect of the quercetin aglycon in the pretreatment with the 3T3 mouse fibroblast cell system [54].

## 4. Conclusions

Quercetin, a member of the flavonoids family and one of the most prominent dietary antioxidants, is claimed to exert beneficial health effects. These include protection against various conditions such as osteoporosis, certain forms of cancer, pulmonary and cardiovascular diseases, and premature aging [55]. It is suggested that quercetin’s ability to scavenge highly reactive oxygen and nitrogen species, such as peroxynitrite and the hydroxyl radical, is involved in these possible beneficial health effects. To counteract peri-implant disease, which can be caused by ROS overproduction, the synthesis of an implant functionalized with the flavonol was achieved. Chemical characterization allowed us to state that the acidic condition of the sol–gel synthesis addressed the preparation of hybrid materials in which C-ring structural features of quercetin were modified. The quercetin derivative retained the *o*-catecholic function determinant for antioxidant capability.

Data obtained for this new hybrid material suggest that it can combine the features required for a high-quality biomaterial and important antioxidant effectiveness, causing an *in vitro* suppression of dehydrogenases activity <25%, which is closely related to only mild cell function suppression. On the basis of these considerations, this work forms the basis of a new research field regarding components of glass ionomer cement with intrinsic antioxidant properties as an alternative effective strategy for preventing the onset of peri-implant diseases.
